# Redox Imbalance and Coronary Complexity Linking Oxidative Stress Biomarkers to SYNTAX II Score in Acute Coronary Syndrome

**DOI:** 10.3390/biomedicines14071489

**Published:** 2026-06-30

**Authors:** Ramazan Düz, Fethullah Kayan, Salih Çibuk, Mihriban Elçiçek, Abdullah Özçelik

**Affiliations:** 1 Department of Cardiology, Faculty of Medicine, Yüzüncü Yıl University, Van 65000, Turkey; ramazanduz054@gmail.com; 2 Department of Cardiology, Gazi Yasargil Training and Research Hospital, University of Health Sciences, Diyarbakır 21070, Turkey; 3 Vocational School of Health Services, Yüzüncü Yıl University, Van 65000, Turkey; salihcibuk@yyu.edu.tr; 4 Department of Nutrition and Dietetics, Tekirdağ Namık Kemal University, Tekirdag 59030, Turkey; mihribanelcicek@gmail.com; 5 Department of Cardiology, Mardin Training and Research Hospital, Mardin 47100, Turkey; drabdullahozcelik@gmail.com

**Keywords:** acute coronary syndrome, oxidative stress, SYNTAX II score, atherosclerosis, CAD severity

## Abstract

**Background:** Lipoprotein metabolism, inflammation, and oxidative stress (OS) play interconnected roles in the pathogenesis of acute coronary syndrome (ACS). However, the relationship between oxidative stress biomarkers and coronary artery disease (CAD) complexity, particularly as assessed by the SYNTAX II score, remains incompletely understood. This study aimed to evaluate serum levels of oxidative stress–related biomarkers and to investigate their association with the SYNTAX II score in patients with newly diagnosed ACS. **Methods:** This was a retrospective, single-center observational study. A total of 60 patients with newly diagnosed ACS who underwent percutaneous coronary intervention (PCI) or coronary artery bypass grafting (CABG) were consecutively enrolled. Serum levels of superoxide dismutase (SOD), advanced oxidation protein products (AOPP), glutathione, catalase, and malondialdehyde (MDA) were measured using ELISA methods. Associations between oxidative stress markers and the SYNTAX II score were analyzed using correlation and regression analyses. **Results:** Median levels of SOD, AOPP, and glutathione were 261.0 (244.18–324.86) U/L, 23.81 (22.62–25.80) ng/mL, and 6.08 (5.28–11.46) ng/mL, respectively. Mean catalase and MDA levels were 339.07 ± 44.81 pg/mL and 0.66 ± 0.07 mmol/L. The SYNTAX II score was positively correlated with SOD, AOPP, glutathione, catalase, and MDA, as well as with age, female sex, and potassium levels, while it was negatively correlated with hemoglobin, creatinine clearance, and left ventricular ejection fraction (all *p* < 0.05). Patients with higher SYNTAX II scores demonstrated elevated levels of both oxidant and antioxidant biomarkers. **Conclusions:** Oxidative stress-related biomarkers were associated with the SYNTAX II score in patients with newly diagnosed ACS.

## 1. Introduction

Atherosclerosis is a chronic inflammatory condition involving the inner walls of arteries. It is characterized by sub-endothelial deposition of modified lipoproteins, proliferation and accumulation of inflammatory factors and immune cells, and development of fibrous tissue/plaques within the vessel wall, leading to decreased blood flow [1]. Atherosclerosis can result in acute coronary syndrome (ACS), coronary artery disease (CAD), peripheral artery disease (PAD), ischemia, stroke or sudden cardiac death (SCD), depending on which arteries are affected [2]. The term ACS describes a series of severe cardiac signs and symptoms associated with coronary atherosclerotic plaques, leading to incomplete or complete coronary occlusion [3]. The best-recognized manifestations of ACS are unstable angina (UA), non-ST elevation myocardial infarction (NSTEMI), and ST-segment elevation myocardial infarction (STEMI) [4]. As ACS continues to be one of the leading causes of morbidity and mortality worldwide, the development of additional myocardial injury biomarkers and imaging studies is crucial for early detection, appropriate management, and favorable outcomes in patients with ACS. Although the exact pathogenesis of ACS has not been fully elucidated, it is well recognized that lipoprotein dysfunctions, inflammation and oxidative stress (OS) are associated with the development and progression of ACS [5,6]. Therefore, biomarkers reflecting OS-related pathways may vary in ACS patients.

The ‘Synergy between percutaneous coronary intervention with Taxus and Cardiac Surgery Study’ (SYNTAX) scoring system is one of the most widely accepted detailed coronary angiographic (CAG) tools. It assesses the severity and complexity of ACS based on anatomical characteristics of the coronary artery, thereby aiding physicians in determining the optimal revascularization approach [7]. However, it is well established that both clinical and anatomical determinants are needed to appropriately stratify the risk of patients undergoing coronary revascularization. Thus, to overcome the pitfalls of the SYNTAX score, the SYNTAX II score was developed to integrate anatomical characteristics with relevant clinical variables [8]. Six clinical parameters, including age, female sex, left ventricular ejection fraction (LVEF), creatinine clearance and the presence/absence of chronic obstructive pulmonary disease or peripheral artery disease, and two anatomical variables (SYNTAX score and unprotected left main coronary artery disease) are used to obtain the SYNTAX II score [8]. This score may predict clinical outcomes in patients with ACS and classify the risk of ACS patients with coronary revascularization. However, despite their pathophysiological significance, few studies have focused on the relationships between OS parameters and the SYNTAX II score in ACS patients, and interestingly, these studies have reported inconclusive findings [9,10]. Despite accumulating evidence linking oxidative stress to ACS pathogenesis, comprehensive studies simultaneously examining multiple oxidative stress biomarkers in relation to the SYNTAX II score—which integrates both anatomical and clinical variables—remain limited, particularly in newly diagnosed ACS cohorts.

As such, the aim of the present study was to determine serum levels of OS-related parameters (including both oxidants and antioxidants) in patients diagnosed with ACS and to assess potential relationships between OS and the SYNTAX II score in the study population. We hypothesized that patients with higher SYNTAX II scores would demonstrate greater oxidative stress burden, characterized by elevated oxidant markers and depleted antioxidant parameters. To reduce the influence of major metabolic and inflammatory confounders on oxidative stress parameters, patients with conditions known to markedly alter redox balance were excluded from the study population.

## 2. Methods

### 2.1. Patient Allocation and Selection

This retrospective cohort study was conducted between August 2022 and December 2022 in the Cardiology Department of Van Yüzüncü Yıl University Faculty of Medicine, Van, Turkey. Sixty patients who were newly diagnosed with ACS and underwent percutaneous coronary intervention (PCI) or coronary artery bypass grafting (CABG) were consecutively enrolled in the study.

Participants younger than 20 years of age, those with acute or chronic infections, malignancy, chronic inflammatory conditions, obesity, excessive alcohol consumption, and substance abuse were excluded. Pregnant females and those within 1 year postpartum, patients with medical conditions that could affect lipid metabolism (such as diabetes mellitus (DM), impaired glucose tolerance, chronic renal disease or chronic liver disease and thyroid disorders), those with PCI failure, those with cognitive impairment, and those with incomplete clinical or angiographic data were also excluded from the study. None of the participants had been administered lipid-lowering therapy (drug usage or lifestyle changes) or antioxidant treatment. None of the participants followed any special diet.

This study did not include a healthy control group, as our primary objective was to assess the relationship between OS-parameters and disease severity (SYNTAX II score) within an ACS population, rather than to compare OS levels between diseased and healthy states. This approach is consistent with similar angiographic scoring studies examining biomarker-severity relationships in ACS cohorts. The focus on intra-disease heterogeneity allows for clinically relevant stratification of ACS patients based on their oxidative stress burden relative to CAD complexity.

### 2.2. Ethics

All research procedures were evaluated and accepted by the Clinical Research Ethics Committee of Van Yüzüncü Yıl University Faculty of Medicine (Decision date: 30 March 2022, decision no: 09) and were conducted in agreement with the ethical standards specified in the Declaration of Helsinki.

### 2.3. Definitions

The ACS definition included the detection of STEMI, NSTEMI, and UA. Acute myocardial infarction (AMI) was defined by the presence of elevated cardiac biomarkers, such as troponins, and at least one of the following: new ischemic characteristic electrocardiogram changes, symptoms associated with angina, alterations in the function of viable myocardium on imaging, or identification of coronary thrombus by invasive CAG. UA-pectoris was diagnosed with the same clinical presentation as AMI, without elevation of cardiac markers. “Newly diagnosed ACS” was defined as patients presenting with their first episode of ACS (STEMI, NSTEMI, or UA) who had no prior history of diagnosed CAD, previous myocardial infarction, or coronary revascularization procedures. All patients underwent CAG within 24 h of admission for acute cardiac symptoms.

### 2.4. Clinical Data Collection

Demographic and clinical characteristics, including age, sex, body mass index (BMI) values, family history, concomitant disorders, echocardiographic and angiographic data on admission, and current medication, were obtained from patients’ medical files. BMI was calculated as weight divided by height squared (kg/m^2^). Systolic and diastolic blood pressure (BP) measurements on admission were recorded. The presence of CAD in a first-degree relative was evaluated as a family history of CAD.

Transthoracic echocardiographic examination of all patients was performed using a cardiovascular ultrasound system (Vivid S5, GE Vingmed Ultrasound AS, Horten, Norway). LVEF was calculated using the apical biplane modified Simpson’s rule to determine left ventricular (LV) systolic function. LV systolic dysfunction was defined as LVEF < 50%. All conventional echocardiographic examinations were performed by two experienced cardiologists who were blinded to patient status and according to the standards of the American Society of Echocardiography.

The patients underwent CAG within 24 h using the standard techniques through the femoral or radial artery. The decision for PCI, CABG or medical therapy was made by a cardiac team consisting of two cardiologists and a cardiovascular surgeon. All PCIs applied in suitable patients were performed according to routine clinical practices, and the option between drug-eluting stent or bare-metal stent alternatives was left to operator discretion. Stenting of the infarct-related artery was successfully performed in all patients.

The SYNTAX II score was performed by two experienced interventional cardiologists and calculated for each patient using an online calculator (version 2.28; available at www.syntaxscore.org). To obtain an anatomical-based SYNTAX score, each coronary artery showing ≥50% luminal stenosis in a vessel of at least 1.5 mm in diameter was scored separately. They were then pooled together to provide the overall SYNTAX score calculated. Calculation of the SYNTAX score is subjected to the assessment of anatomical variables: coronary dominance, location of the diseased coronary artery vessel and segment, and the presence of bifurcation or trifurcation lesion, total occlusion, severe tortuosity, calcification, aorto-ostial lesion, thrombus, length of lesion (>20 mm), and diffuse/small vessel disease.

### 2.5. Laboratory Data Collection

After overnight fasting within the first 24 h after hospitalization, blood samples were drawn from the antecubital vein and were centrifuged at 1500× *g* for 10 min to separate the serum. Serum total cholesterol, triglyceride, and fasting blood glucose were measured with photometric methods on an Abbott Architect c8000 analyzer with commercially available kits (Abbott Laboratories, Texas, IL, USA). Serum low-density lipoprotein cholesterol (LDL-C) levels were calculated using the Friedewald formula [11]. Troponin I was measured with chemiluminescent enzyme immunoassay on a Cobas E 601 device (Roche Diagnostics GmbH, Mannheim, Germany). Serum superoxide dismutase (SOD), advanced oxidation protein products (AOPP), glutathione, catalase and malondialdehyde (MDA) were determined using Enzyme-Linked Immunosorbent Assay (ELISA) kits (AFG Bioscience, Arlington Heights, IL USA, for AOPP and MDA; LifeSpan Biosciences, Seattle, WA, USA, for catalase; and BT Lab, Bioassay Technology Laboratories, Shanghai, China, for SOD and glutathione) according to the manufacturers’ instructions. Minimum detection limits were 3 U/L for SOD, 1.3 ng/mL for AOPP, 0.1 ng/mL for glutathione, 15.625 pg/mL for catalase, and 0.3 mmol/L for MDA.

### 2.6. Statistical Analysis

Because of the retrospective and exploratory design of the study, no formal a priori sample size calculation was performed. The study sample consisted of all eligible consecutive patients who met the inclusion and exclusion criteria during the predefined study period. Therefore, the findings, particularly those derived from multivariable analyses, should be interpreted as exploratory and hypothesis-generating. The statistical analysis was conducted using IBM SPSS Statistics Version 25.0 for Windows (IBM Corp., Armonk, NY, USA). Normality of variables was assessed using histograms and Q-Q plots. Continuous variables were expressed as mean ± standard deviation or median (1st quartile–3rd quartile), according to distribution characteristics, while categorical variables were presented as frequency (percentage). Between-group comparisons were performed using Student’s *t*-test or Mann–Whitney U test for continuous variables, depending on normality of distribution, and chi-square test or Fisher’s exact test for categorical variables. Pearson, Spearman, or point bi-serial correlation coefficients were used to evaluate associations between variables. Multivariable logistic regression analysis (forward conditional selection method) was performed to determine factors independently associated with intermediate and high SYNTAX II score. Variables demonstrating *p* < 0.10 in the univariable analyses, together with clinically relevant variables, were considered candidates for multivariable logistic regression. To reduce the risk of model overfitting in view of the relatively small sample size, the final model was constructed using a forward conditional selection procedure. Only variables retained by the forward conditional selection procedure were included in the final regression model to minimize model complexity and reduce the risk of overfitting. Multicollinearity among variables included in the regression analysis was assessed using correlation matrices, variance inflation factor (VIF), and tolerance statistics prior to model construction. A VIF value < 5 and tolerance > 0.20 were considered indicative of the absence of significant multicollinearity. A *p*-value < 0.05 was considered statistically significant.

## 3. Results

A total of 60 patients with ACS were included in the study. The patients consisted of 49 (81.67%) men and 11 (18.33%) women, with a mean age of 62.3 ± 10.5 years. The mean SYNTAX II score of the subjects was 25.23 ± 7.94. Twenty-four (40.00%) subjects had a low (≤22) SYNTAX score, 28 (46.67%) subjects had an intermediate (23–32) score, and 8 (13.33%) subjects had a high (≥33) II score. Age (*p* < 0.001) and female percentage (*p* = 0.037) were significantly higher in the intermediate and high SYNTAX II score group, while LVEF (*p* = 0.026) was significantly higher in the low SYNTAX II score group (Table 1).

**Figure 1:**
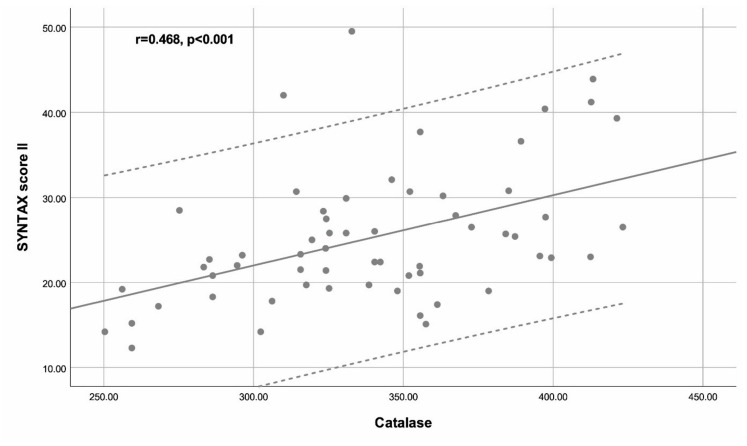
Scatter plot illustrating the correlation between catalase levels and SYNTAX II score.

**Figure 2:**
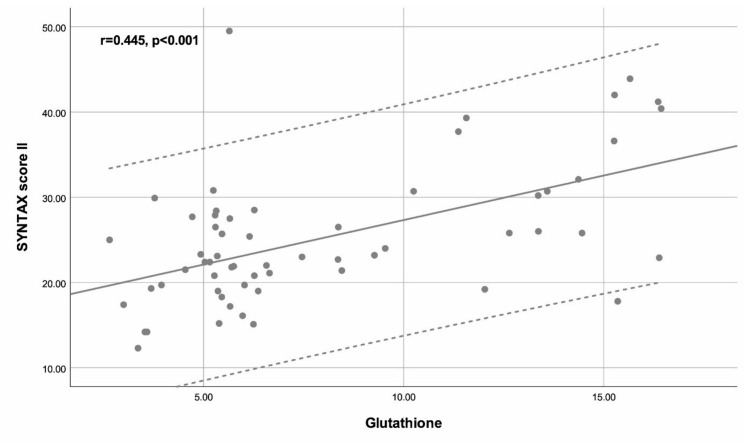
Scatter plot illustrating the correlation between glutathione levels and SYNTAX II score.

**Figure 3:**
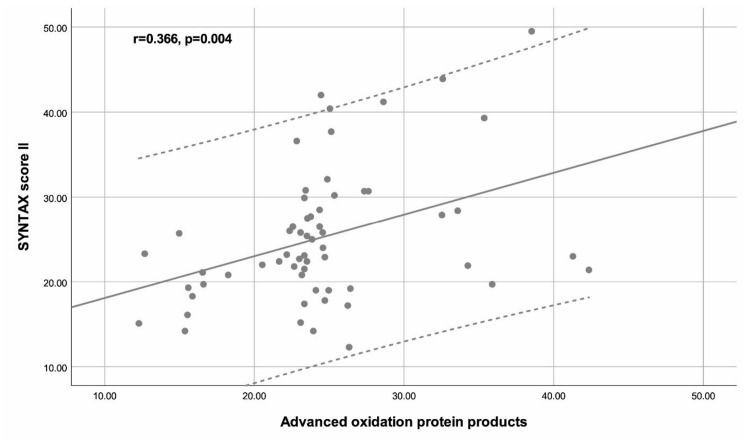
Scatter plot illustrating the correlation between advanced oxidation protein products (AOPP) and SYNTAX II score.

**Figure 4:**
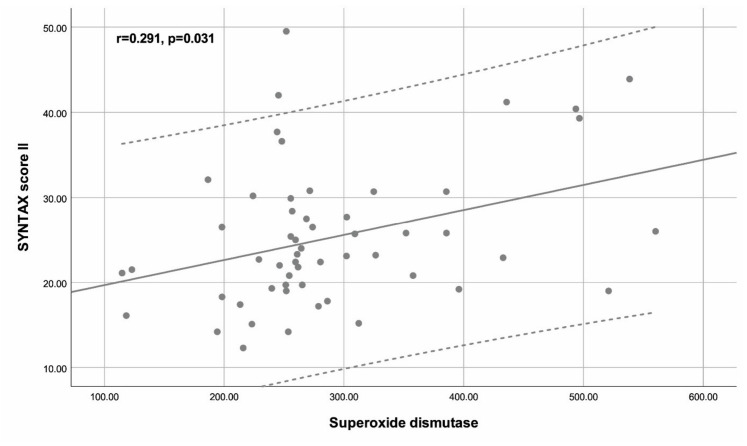
Scatter plot illustrating the correlation between superoxide dismutase (SOD) and SYNTAX II score.

Catalase (*p* < 0.001) (Figure 1), glutathione (*p* < 0.001) (Figure 2), advanced oxidation protein products (*p* = 0.004) (Figure 3), and SOD (*p* = 0.031) (Figure 4) were significantly higher in the intermediate and high SYNTAX II score group (Table 2).

**Figure 5:**
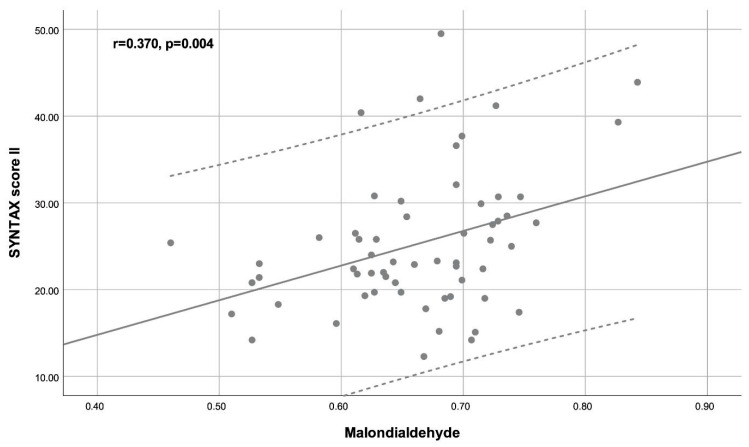
Scatter plot illustrating the correlation between malondialdehyde (MDA) and SYNTAX II score.

The results of correlation analyses were presented in Table 3. The SYNTAX II score was positively correlated with catalase (Figure 1), glutathione (Figure 2), AOPP (Figure 3), SOD (Figure 4), MDA (Figure 5), age, and female sex, whereas it was negatively correlated with LVEF (all, *p* < 0.05). SOD was also positively correlated with systolic BP and negatively with LVEF (*p* = 0.007 and *p* = 0.006, respectively). AOPP was found to correlate positively with age (*p* = 0.008). Glutathione was positively correlated with age and negatively correlated with LVEF (*p* = 0.004 and *p* = 0.004, respectively). Catalase level was positively correlated with age and the presence of Cx-vessel disease.

Nine candidate variables (age, sex, LVEF, potassium, creatinine clearance, SOD, glutathione, catalase, MDA) demonstrating *p* < 0.10 in the univariable analyses, together with clinically relevant variables, were entered into the forward conditional multivariable logistic regression model.

Multivariable logistic regression analysis results revealed that high age (OR: 1.085, 95% CI: 1.011–1.165, *p* = 0.024) and high catalase levels (OR: 1.020, 95% CI: 1.003–1.038, *p* = 0.024) were independently associated with an intermediate and high SYNTAX score. Other variables included in the analysis—sex (*p* = 0.056), LVEF (*p* = 0.088), potassium (*p* = 0.208), creatinine clearance (*p* = 0.071), SOD (*p* = 0.074), glutathione (*p* = 0.053) and MDA (*p* = 0.368)—were found to be non-significant (Table 4).

Multicollinearity diagnostics demonstrated acceptable collinearity statistics among the variables included in the regression model. All variance inflation factor (VIF) values were below 5, and tolerance values were above 0.20, suggesting no significant multicollinearity.

## 4. Discussion

This study suggests that there may be associations between SYNTAX II score and multiple OS parameters in newly diagnosed ACS patients. Our findings confirmed that patients with intermediate and high SYNTAX II scores had significantly elevated OS markers compared to those with low scores.

The proper functioning of biological systems depends on maintaining the balance of pro-oxidants and antioxidants [12]. Under physiological conditions, reactive oxygen/nitrogen species (ROS/RNS) are produced at moderate concentrations and play a crucial role in inflammatory responses, cell signaling and growth, apoptosis, and changes in vascular tone as well as in oxidation of LDL-C [13]. With the increased production of ROS/RNS and/or decreased capacity of the organism to counteract the action of ROS/RNS, the oxidant–antioxidant balance is disturbed, leading to OS [14]. OS is a well-known contributor to the occurrence and progression of many disorders, including cancer, neurodegenerative and inflammatory disorders, and cardiovascular disease (CVD) [14], and it is also a key mechanism in the pathogenesis of atherosclerosis, in concert with pro-inflammatory signaling and expression of cytokines/chemokines. As such, OS is a well-recognized traditional cardiovascular risk factor that is also associated with DM, hypertension, dyslipidemia, age and sex [15]. Early atherosclerosis is associated with endothelial damage, resulting in the subsequent infiltration and accumulation of LDL-C into the sub-endothelial space [16]. LDL-C are oxidized by a complex set of biochemical reactions involving enzymes and free radicals in the endothelium [17]. Initial endothelial damage and inflammatory responses lead to the accumulation of foam cells [18]. These growth factors and ROS promote smooth muscle cell migration and collagen deposition as well as secretion of matrix metalloproteinases, which disrupt the fibrous wall of the atheromatous plaque and the basement membrane of endothelial cells, leading to the progression of atherosclerosis and, ultimately, ACS [19]. Therefore, the underlying relationships between oxidants and antioxidants are critical for disease development and progression and could also be associated with cardiac risk-scoring systems such as the SYNTAX score, and particularly with the SYNTAX II score. Importantly, several clinical variables included in our regression analyses, such as age, sex, LVEF, and creatinine clearance, are also integral components of the SYNTAX II score itself. Therefore, a degree of conceptual overlap between predictor variables and the outcome measure is unavoidable. In addition, oxidative stress markers were correlated with some of these clinical parameters. To address this issue, multicollinearity analyses using VIF and tolerance statistics were performed and did not indicate significant statistical collinearity. Nevertheless, partial collinearity and residual confounding cannot be fully excluded, and the observed associations should therefore be interpreted cautiously. In addition, the relatively small sample size may have contributed to unstable coefficient estimates; therefore, the reported odds ratios should be interpreted cautiously until validated in larger independent cohorts. Accordingly, future studies incorporating internal and external validation procedures are warranted before these findings can be translated into clinical risk prediction models. While routine lipid parameters and inflammatory cell counts were measured, our analysis focused specifically on direct oxidative stress biomarkers (SOD, AOPP, glutathione, catalase, MDA) as these provide a more direct assessment of oxidant–antioxidant imbalance in ACS pathophysiology than indirect inflammatory markers.

In our ACS cohort, we observed elevated levels of both oxidant markers (AOPP and MDA) and antioxidant enzymes (glutathione, catalase, and SOD) in patients with higher SYNTAX II scores, reflecting more severe and complex CAD. The observed elevation in antioxidant enzyme levels in patients with higher SYNTAX II scores may reflect an adaptive or compensatory response to increased oxidative stress. However, given the observational, cross-sectional design of the present study, this interpretation remains speculative and no causal or mechanistic relationship can be established.

A simultaneous increase in oxidant and antioxidant biomarkers has been reported in some cardiovascular conditions. One possible explanation is the activation of endogenous antioxidant defense systems in response to increased oxidative stress. However, the present observational cross-sectional study cannot determine whether such a compensatory mechanism truly exists, and this interpretation should therefore be regarded as a hypothesis rather than a demonstrated biological mechanism. This finding should be interpreted with caution, as increased antioxidant levels may not necessarily indicate an effective protective response. Alternatively, increased antioxidant enzyme levels may represent altered biomarker kinetics, differences in oxidative status at the time of sampling, or methodological characteristics of the assays rather than a true compensatory biological response. In addition, it should be noted that ELISA-based measurements may not distinguish between reduced and oxidized forms of certain biomarkers, such as glutathione, potentially influencing the observed results. The major antioxidant systems in the vascular wall include SOD, catalase, glutathione peroxidase, parooxanases and NO synthases [20]. The primary way in which vascular cells protect themselves against superoxide is through SOD, which converts it into hydrogen peroxide. Afterward, the hydrogen peroxide is broken down by catalases, glutathione peroxidases, and thioredoxins [5]. By their direct effect in converting hydrogen peroxide, which is a harmful by product of various metabolic processes, into water and oxygen, catalases have been shown to improve atherosclerosis in mouse models on a high-fat diet [18]. Previous studies reported that glutathione is the main intracellular antioxidant and acts as a cofactor in many conjugation detoxification reactions and may protect against CVD [21]. MDA is considered a biomarker of lipid peroxidation, which is often related to CVD [21]. AOPP is a product of the oxidation of plasma proteins by exposure to ROS in the bloodstream, and its levels are closely associated with OS response [22,23,24]. Similar to our results, Aladağ et al. recently demonstrated in 50 NSTEMI patients, 50 STEMI patients and 50 healthy subjects that ACS patients had higher serum MDA and ischemia-modified albumin (IMA) levels and lower SOD and catalase activities [25]. Haque et al. showed decreased serum glutathione, total antioxidant capacity and antioxidant vitamins (C, D and alpha-tocopherol levels) in 138 ACS patients compared to 134 non-ACS controls [26]. In a study by Karakayalı et al., patients with UA, NSTEMI, and STEMI were analyzed for both antioxidant and oxidant parameters. The results showed that oxidant parameters such as protein carbonyl and MDA increased progressively, while antioxidant parameters such as vitamin C, vitamin A, and carotenoids decreased gradually, from controls to UA, NSTEMI, and STEMI [27].

Our findings support an association between oxidative stress-related biomarkers and CAD complexity in ACS patients. However, the present study design does not allow conclusions regarding causality or direct mechanistic involvement.

These findings are consistent with previous evidence suggesting that ROS production contributes to endothelial dysfunction, plaque progression, and ultimately acute coronary events. Nevertheless, previous studies have reported heterogeneous findings regarding antioxidant responses in ACS, with some demonstrating decreased antioxidant capacity. These discrepancies may be related to differences in patient populations, the timing of blood sampling after symptom onset, assay methodology, and the relative proportions of STEMI, NSTEMI, and unstable angina among study populations. Additionally, decreased levels of glutathione, catalase, and SOD may be a result of their heightened utilization to eliminate ACS-induced ROS.

Given the important role of OS in ACS, the relationship between OS and the complexity of CAD in patients with ACS is unclear. Various angiographic scoring systems have been developed to quantify the complexity of CAD, including the Gensini and SYNTAX systems [28]. Because a patient’s clinical features have a significant impact on prognosis, the SYNTAX II score was developed, and it is a better predictor of morbidity and mortality than SYNTAX, regardless of clinical presentation [8]. However, few studies have investigated the relationship between OS-related biomolecules and the SYNTAX II score in ACS patients. Çağdaş and colleagues demonstrated in 344 ACS patients treated with PCI that those with a higher SYNTAX II score had a higher C-reactive protein-to-albumin ratio compared to those with a low SYNTAX II score [29], indicating greater levels of systemic inflammation and oxidative stress. Bosnjak et al. showed in 168 patients with suspected CAD who required PCI (n = 64), CABG (n = 57), or no intervention (n = 47) that galectin-3 levels were increased in recipients of coronary intervention. Furthermore, a moderate relationship was found between galectin-3 levels and the SYNTAX II score in the PCI group, but not in the CABG group [10]. Galectin-3 may initiate inflammatory mechanisms by inducing neutrophil superoxide production, triggering OS reactions, and promoting ox-LDL uptake by macrophages, smooth muscle cells, and vascular endothelial cells, leading to atherosclerosis. Çağdaş et al. revealed that the higher SYNTAX and SYNTAX II scores in STEMI patients were related to a high monocyte-to-HDL-C ratio, which is used as a biomarker of inflammation and oxidative stress [30]. In contrast, Sökmen et al. showed no significant relationship between both arylesterase and paraoxonase-1 activities and the SYNTAX II score in 102 STEMI patients [9]. In addition, Ermiş et al. demonstrated no significant relationship between thiol/disulfide and IMA levels and the SYNTAX II score in 142 ACS patients [31]. Salvatore et al. showed that the presence of unprotected left main stenosis and a SYNTAX II score >29 were the only predictors of major adverse cardiac and cerebrovascular events at 1-year clinical follow-up in patients with ACS and severe CAD who had undergone PCI [32]. Similar to their results showing a median SYNTAX II score of 29 (range: 14–59), we found the mean SYNTAX II score to be 25.23 ± 7.94 in our study population. Previous studies have also evaluated oxidative stress parameters in relation to coronary lesion complexity and the SYNTAX score. Vukicevic et al. reported that oxidative stress markers were associated with the SYNTAX score in patients undergoing coronary artery bypass surgery, supporting a relationship between redox imbalance and the anatomical complexity of coronary atherosclerosis. In another study, platelet activation/reactivity indices and oxidative stress parameters were assessed in patients undergoing different coronary artery bypass grafting strategies, further emphasizing the interaction between oxidative stress, platelet biology, and coronary disease burden. Moreover, low paraoxonase-1 activity together with the SYNTAX score was reported to predict postoperative complications after coronary artery surgery. Collectively, these studies support the concept that oxidative stress-related pathways are closely linked to coronary atherosclerotic burden and cardiovascular risk. Our findings extend these observations by demonstrating significant associations between multiple oxidant and antioxidant biomarkers and the SYNTAX II score in a cohort of newly diagnosed ACS patients [33,34,35].

Our data indicates that OS-related parameters were related to anatomical and clinical CAD severity—as characterized by the SYNTAX II score. Identifying OS parameters in ACS patients may enable earlier evaluation of patients at higher risk of experiencing adverse events. Management strategies targeting ROS/RNS production, antioxidant pathways, and inhibition of oxidized LDL-cholesterol generation may reduce OS, thereby improving various characteristics of patients with atherosclerosis and ACS. We also demonstrated a relationship between the SYNTAX II score and several clinical parameters, consistent with the multifactorial nature of CAD severity. Age demonstrated the strongest correlation with the SYNTAX II score, which is expected because age is an integral component of the SYNTAX II algorithm. Therefore, oxidative stress biomarkers should be interpreted as complementary biological correlates of CAD complexity rather than primary determinants of the SYNTAX II score. Similar to our results, Çağdaş et al. also showed an association between decreased hemoglobin values and an increased SYNTAX II score [29]. However, decreased hemoglobin values and increased potassium levels could be associated with advanced age, reduced GFR and lower LVEF, which are utilized in SYNTAX II calculation. Therefore, these relationships should be assessed with caution, and further studies must be performed to identify independently associated variables. Given the relatively limited sample size and number of events, the multivariable regression findings should be considered exploratory and hypothesis-generating. Future studies involving larger and more representative cohorts, together with alternative analytical approaches designed to minimize conceptual overlap between predictor variables and the SYNTAX II score, may further clarify the independent contribution of oxidative stress biomarkers to CAD complexity. The possibility of model overfitting and unstable coefficient estimation cannot be completely excluded.

Several important confounders warrant consideration in interpreting our findings. The sex imbalance between SYNTAX II groups, with a higher proportion of women in the intermediate/high score category, may partially influence OS marker levels given known sex-related differences in redox physiology. Age—a significant independent predictor in our multivariable model—is itself associated with both increased OS and higher SYNTAX II scores. While our multivariable analysis controlled for sex and age, residual confounding from unmeasured variables such as smoking status, dietary antioxidant intake, and medication use cannot be excluded. The exclusion of patients with diabetes, renal disease, and inflammatory conditions, while reducing confounding from these specific factors, may limit generalizability to the broader ACS population in whom these comorbidities commonly coexist. In real-world ACS populations, comorbid conditions such as diabetes mellitus, chronic kidney disease, obesity, and chronic inflammatory disorders are highly prevalent and are themselves strongly associated with increased oxidative stress and endothelial dysfunction. Therefore, the exclusion of these patient groups may have resulted in a more selected study population with lower biological heterogeneity. While this methodological approach strengthened the internal validity of the study by reducing confounding effects on oxidative stress biomarkers, it was achieved at the expense of external validity, thereby limiting the generalizability of our findings to routine real-world ACS populations. Accordingly, our results should primarily be interpreted as hypothesis-generating and require confirmation in larger prospective studies involving more representative ACS cohorts.

Recent narrative and systematic reviews suggest that, beyond traditional biomarkers, several emerging inflammatory and cardiovascular biomarkers may improve risk stratification in patients with acute coronary syndrome. Biomarkers such as interleukin-6 (IL-6), interleukin-18 (IL-18), soluble ST2 (sST2), galectin-3, growth differentiation factor-15 (GDF-15), matrix metalloproteinase-9 (MMP-9), and copeptin reflect complementary pathophysiological mechanisms including inflammation, plaque instability, myocardial stress, extracellular matrix remodeling, and endothelial dysfunction, thereby providing prognostic information beyond conventional cardiac biomarkers in selected clinical settings [36,37]. Nevertheless, current evidence indicates that most of these novel biomarkers should be considered complementary rather than replacements for established biomarkers and validated clinical risk scores. Future multimarker approaches integrating oxidative stress biomarkers with inflammatory and myocardial stress markers may improve individualized risk stratification and provide a more comprehensive understanding of ACS pathophysiology [36,37,38].

Our findings add to the growing body of evidence linking oxidative stress-related pathways with coronary artery disease severity and complexity. Previous studies have demonstrated associations between individual oxidative stress markers, antioxidant enzyme activities, platelet activation parameters, and SYNTAX-based angiographic scores. In the present study, we observed significant associations between multiple oxidant (AOPP, MDA) and antioxidant (SOD, glutathione, catalase) biomarkers and SYNTAX II score in patients with newly diagnosed ACS. These findings further support the concept that redox imbalance is closely related to both the anatomical and clinical complexity of coronary artery disease. Nevertheless, given the observational nature of the study, the underlying mechanisms and potential clinical utility of these biomarkers require further investigation in larger prospective studies.

These findings suggest that oxidative stress biomarkers may offer additional biological insight beyond conventional anatomical and clinical risk scores in patients with ACS. However, their potential role in clinical decision-making and risk stratification remains to be clarified. Larger prospective studies incorporating mechanistic and longitudinal assessments are needed to better define the clinical relevance of oxidative stress profiling in this setting.

## 5. Limitations

This study has several limitations that warrant consideration. Given its cross-sectional design, no causal relationship can be inferred between oxidative stress markers and CAD complexity. Therefore, interpretations regarding compensatory antioxidant activation or adaptive redox responses should be considered hypothetical and require confirmation in longitudinal and mechanistic studies. First, the absence of a healthy control group limits our ability to definitively characterize whether OS markers are elevated or depleted compared to normal physiology. However, the primary aim of this study was to evaluate the relationship between oxidative stress parameters and disease severity within an ACS population, rather than to compare biomarker levels between diseased and healthy individuals. Second, the single-center design and relatively small sample size (n = 60) may limit statistical power and generalizability. A limited number of events in the intermediate/high SYNTAX II subgroup may have reduced the statistical power of the multivariable regression analysis and increased susceptibility to model overfitting. Although the number of covariates was restricted to improve model stability, the regression results should be interpreted as exploratory rather than definitive. Larger prospective multicenter studies are required to validate these findings. Third, the significant sex imbalance between SYNTAX II groups (95.8% vs. 72.2% male) and the small number of female participants (n = 11) preclude robust sex-specific analyses and represent a potential source of residual confounding despite statistical adjustment. Fourth, smoking status, an important determinant of oxidative stress and coronary artery disease severity, was not systematically recorded in the retrospective medical records and therefore could not be included in the statistical analysis. This represents an important unmeasured confounder. Fifth, our strict exclusion criteria—including diabetes mellitus, chronic kidney disease, obesity, and inflammatory disorders—may substantially limit the generalizability of our findings to routine, real-world ACS populations, in whom these comorbidities are highly prevalent. However, these criteria were intentionally applied to minimize potential confounding effects on oxidative stress pathways and to better isolate the relationship between oxidative stress biomarkers and CAD complexity. Sixth, oxidative stress markers were measured at a single time point within 24 h of admission. Since oxidative stress is a dynamic process during ACS and after revascularization, serial measurements at predefined post-PCI intervals would have provided a more accurate characterization of temporal changes in oxidant–antioxidant balance, including peak responses and recovery patterns. Therefore, our findings reflect only a single-time-point association between oxidative stress biomarkers and SYNTAX II score, rather than longitudinal oxidative stress dynamics. Seventh, oxidative stress is a complex and dynamic biological process that cannot be fully characterized by a limited panel of biomarkers obtained at a single time point. We did not measure oxidized LDL, total antioxidant capacity, glutathione redox status (GSH/GSSG ratio), inflammatory cytokines, endothelial dysfunction markers, or high-sensitivity CRP. Furthermore, serial biomarker measurements were not available. Future studies incorporating these biomarkers together with serial measurements may provide more comprehensive mechanistic insights into the relationship between oxidative stress and CAD complexity.

Finally, the lack of long-term clinical follow-up prevents assessment of whether OS markers predict cardiovascular outcomes beyond their association with angiographic severity. In addition, comprehensive internal validation procedures, including bootstrap resampling, cross-validation, calibration assessment, and discrimination analysis, were not performed because of the relatively limited sample size and exploratory nature of the study. Consequently, the stability and generalizability of the regression model cannot be fully established, and the reported odds ratios should therefore be interpreted cautiously until validated in larger independent cohorts.

## 6. Conclusions

Oxidative stress-related biomarkers were associated with the SYNTAX II score in patients with newly diagnosed ACS, suggesting a close relationship between redox imbalance and coronary artery disease complexity. These findings support the potential role of oxidative stress profiling as a complementary tool for risk assessment in ACS and warrant further investigation in larger prospective studies.

## Figures and Tables

**Table 1:** Demographic and clinical characteristics of the participants according to SYNTAX II score.

		SYNTAX II Score		
	Total (n = 60)	Low (n = 24)	Intermediate & High (n = 36)	*p*
Age, years	62.32 ± 10.52	56.46 ± 8.14	66.22 ± 10.19	**<0.001**
Sex				
Male, (n, %)	49 (81.67%)	23 (95.83%)	26 (72.22%)	**0.037**
Female, (n, %)	11 (18.33%)	1 (4.17%)	10 (27.78%)	
Body mass index, kg/m^2^	27.00 ± 2.62	26.96 ± 2.81	27.03 ± 2.52	0.921
Family history, (n, %)	32 (53.33%)	13 (54.17%)	19 (52.78%)	1.000
The presence of hypertension, (n, %)	46 (76.67%)	17 (70.83%)	29 (80.56%)	0.575
Systolic blood pressure, mmHg	137.40 ± 15.03	134.33 ± 14.47	139.44 ± 15.25	0.199
Diastolic blood pressure, mmHg	80.93 ± 7.51	80.25 ± 5.99	81.39 ± 8.43	0.543
Affected vessel, (n, %)				
LAD	25 (41.67%)	9 (37.50%)	16 (44.44%)	0.789
RCA	22 (36.67%)	11 (45.83%)	11 (30.56%)	0.353
Cx	14 (23.33%)	4 (16.67%)	10 (27.78%)	0.493
Other	11 (18.33%)	5 (20.83%)	6 (16.67%)	0.741
Requiring coronary artery by-pass graft, (n, %)	2 (3.33%)	1 (4.17%)	1 (2.78%)	1.000
Left ventricular ejection fraction, %	55 (50–60)	55 (55–60)	52.5 (45–57.5)	**0.026**

LAD: Left anterior descending artery; RCA: Right coronary artery; Cx: Circumflex coronary artery. Data are given as mean ± standard deviation or median (1st quartile–3rd quartile) for continuous variables according to normality of distribution and as frequency (percentage) for categorical variables.

**Table 2:** Laboratory findings of the participants according to SYNTAX II score.

		SYNTAX II Score		
	Total (n = 60)	Low (n = 24)	Intermediate & High (n = 36)	*p*
Total cholesterol, mg/dL	165.60 ± 35.42	170.33 ± 28.82	162.44 ± 39.28	0.403
Triglyceride, mg/dL	143.5 (100.5–198.5)	143.5 (102.5–210)	141 (99.5–186)	0.411
LDL-C, mg/dL	98.15 ± 32.34	95.58 ± 30.28	99.86 ± 33.95	0.619
Fasting blood glucose, mg/dL	110.5 (96.5–154.0)	106.2 (94–128.5)	120 (106–159)	0.126
Peak Troponin I, pg/mL	0.105 (0.007–1.194)	0.105 (0.007–3.479)	0.130 (0.007–1.152)	0.634
Creatinine clearance, mL/min	87.91 ± 26.32	100.62 ± 19.62	79.44 ± 27.03	**0.002**
Superoxide dismutase, U/L	261.00 (244.18–324.86)	251.65 (213.35–278.74)	271.50 (255.62–351.84)	**0.031**
Advanced oxidation protein products, ng/mL	23.81 (22.62–25.80)	23.26 (16.58–25.61)	24.36 (23.22–26.35)	0.004
Glutathione, ng/mL	6.08 (5.28–11.46)	5.68 (4.24–6.31)	8.36 (5.30–13.48)	**<0.001**
Catalase, pg/mL	339.07 ± 44.81	314.07 ± 39.24	355.74 ± 40.73	**<0.001**
Malondialdehyde, mmol/L	0.66 ± 0.07	0.64 ± 0.07	0.68 ± 0.07	**0.026**

LDL-C: Low-density lipoprotein cholesterol. Data are given as mean ± standard deviation or median (1st quartile–3rd quartile) for continuous variables according to normality of distribution and as frequency (percentage) for categorical variables.

**Table 3:** Correlations between SYNTAX II score, oxidative stress markers and other variables.

		SYNTAX Score II	Superoxide Dismutase	Advanced Oxidation Protein Products	Glutathione	Catalase	Malondialdehyde
SYNTAX II score	*r*	-	**0.291**	**0.366**	**0.445**	**0.468**	**0.370**
	*p*	-	**0.031**	**0.004**	**<0.001**	**<0.001**	**0.004**
Age	*r*	**0.715**	0.103	**0.342**	**0.371**	**0.490**	0.154
	*p*	**<0.001**	0.456	**0.008**	**0.004**	**<0.001**	0.239
Sex, female	*r*	**0.407**	0.136	0.093	0.230	0.222	0.217
	*p*	**0.001**	0.322	0.478	0.077	0.088	0.096
Systolic blood pressure	*r*	0.118	**0.363**	0.139	0.128	0.175	−0.104
	*p*	0.368	**0.007**	0.290	0.331	0.182	0.431
Affected vessel, Cx	*r*	0.114	0.160	0.176	0.082	**0.280**	0.099
	*p*	0.385	0.242	0.178	0.534	**0.030**	0.450
Left ventricular ejection fraction	*r*	**−0.386**	**−0.363**	−0.201	**−0.368**	−0.124	−0.158
	*p*	**0.002**	**0.006**	0.123	**0.004**	0.346	0.228

Cx: Circumflex coronary artery; *r*: Correlation coefficient.

**Table 4:** Significant factors independently associated with intermediate and high (>22) SYNTAX score: multivariable logistic regression analysis.

	β Coefficient	Standard Error	*p*	Exp (β)	95.0% CI for Exp (β)	
Age, years	0.082	0.036	**0.024**	1.085	1.011	1.165
Catalase, pg/mL	0.020	0.009	**0.024**	1.020	1.003	1.038
Constant	−11.294	3.346	0.001			

Nagelkerke R^2^ = 0.379; CI: Confidence interval.

## Data Availability

The data and materials used in this study are available upon request from the authors. Interested researchers may contact the corresponding author to discuss data access, subject to ethical and privacy considerations.

## Abbreviations

OS	Oxidative Stress
ACS	Acute Coronary Syndrome
SYNTAX	Synergy Between Percutaneous Coronary İntervention with Taxus and Cardiac Surgery Study
PCI	Percutaneous Coronary Intervention
CABG	Coronary Artery Bypass Grafting
CABGO	Coronary Artery Bypass Grafting Operation
SOD	Superoxide Dismutase
AOPP	Advanced Oxidation Protein Products
MDA	Malondialdehyde
LVEF	Left Ventricular Ejection Fraction
CAD	Coronary Artery Disease
PAD	Peripheral Artery Disease
SCD	Sudden Cardiac Death
UA	Unstable Angina
NSTEMI	Non-ST Elevation Myocardial Infarction
STEMI	ST-Segment Elevation Myocardial Infarction
CAG	Coronary Angiographic
DM	Diabetes Mellitus
AMI	Acute Myocardial Infarction
BMI	Body Mass Index
BP	Blood Pressure
LV	Left Ventricular
LDL-C	Low-Density Lipoprotein Cholesterol
ELISA	Enzyme-Linked Immunosorbent Assay
OR	Odds Ratio
CI	Confidence Interval
ROS	Reactive Oxygen Species
RNS	Reactive Nitrogen Species
CVD	Cardiovascular Disease
IMA	Ischemia-Modified Albumin
HDL-C	High Density Lipoprotein Cholesterol
GSSG	Glutathione Disulfide
GSH	Glutathione
GFR	Glomerular Filtration Rate
